# Guillain-Barré Syndrome and Preceding Infection with *Campylobacter*, Influenza and Epstein-Barr Virus in the General Practice Research Database

**DOI:** 10.1371/journal.pone.0000344

**Published:** 2007-04-04

**Authors:** Clarence C. Tam, Sarah J. O'Brien, Irene Petersen, Amir Islam, Andrew Hayward, Laura C. Rodrigues

**Affiliations:** 1 Infectious Disease Epidemiology Unit, Department of Epidemiology and Population Health, London School of Hygiene and Tropical Medicine, London, United Kingdom; 2 Environmental and Enteric Diseases Department, Health Protection Agency Centre for Infections, London, United Kingdom; 3 Division of Medicine and Neuroscience, School of Medicine, University of Manchester, Salford, United Kingdom; 4 Department of Primary Care and Population Sciences, University College, London, London, United Kingdom; US Naval Medical Research Center Detachment/Centers for Disease Control, United States of America

## Abstract

**Background:**

A number of infectious agents have previously been suggested as risk factors for the development of Guillain-Barré syndrome (GBS), but robust epidemiologic evidence for these associations is lacking.

**Methods and Findings:**

We conducted a nested case-control study using data from the United Kingdom General Practice Research Database between 1991 and 2001. Controls were matched to cases on general practice clinic, sex, year of birth and date of outcome diagnosis in their matched case. We found positive associations between GBS and infection with *Campylobacter*, Epstein-Barr virus and influenza-like illness in the previous two months, as well as evidence of a protective effect of influenza vaccination. After correction for under-ascertainment of *Campylobacter* infection, the excess risk of GBS following *Campylobacter* enteritis was 60-fold and 20% of GBS cases were attributable to this pathogen.

**Conclusions:**

Our findings indicate a far greater excess risk of GBS among *Campylobacter* enteritis patients than previously reported by retrospective serological studies. In addition, they confirm previously suggested associations between infection due to Epstein-Barr virus infection and influenza-like illness and GBS. Finally, we report evidence of a protective effect of influenza vaccination on GBS risk, which may be mediated through protection against influenza disease, or result from a lower likelihood of vaccination among those with recent infection. Cohort studies of GBS incidence in this population would help to clarify the burden of GBS due to influenza, and any potential protective effect of influenza vaccination.

## Introduction

Guillain-Barré syndrome (GBS) is the most common cause of acute flaccid paralysis in polio-free regions, with incidence estimated at between 0.4 and 4 per 100,000 in different settings [1]. The disease has an autoimmune pathology; following infection, antibodies produced against pathogen surface structures cross-react with nerve ending antigens, leading to neurologic damage. Several pathogens are thought to trigger GBS, primarily *Campylobacter jejuni*. Numerous studies have demonstrated evidence for an association between GBS and preceding *C. jejuni* infection. These studies have mostly relied on serologic evidence of *C. jejuni* infection, reporting infection prevalences of 15% to 66% among GBS cases compared with 0% to 17% in controls [2]–[11], and odds ratios between three and five [3], [6]. However, serologic tests are not specific for recent *C. jejuni* infection. These studies are thus difficult to interpret; seropositivity could indicate recent infection, past infection, or immunity, and the distribution of these is likely to differ between seropositive cases and seropositive controls, leading to biased estimates of the *Campylobacter*-GBS association. A Swedish capture-recapture study reported a GBS incidence of 3.0 per 10,,000 among *Campylobacter* enteritis cases reported to national surveillance, 100 times the incidence of GBS in the general population [12]. Using data from a cohort of patients presenting to primary care, we have previously estimated that for every 10,000 cases of *Campylobacter* enteritis, two cases of GBS occur within the two months following infection, an incidence 77 times greater than that in the general population [13], [14]. Our previous studies have indicated that between nine and 14 percent of GBS cases are attributable to symptomatic *Campylobacter* infection [13], [14], suggesting that asymptomatic infection, or infection with other pathogens, must account for the majority of GBS cases.

Other pathogens suggested to trigger GBS include cytomegalovirus [8], [15], Epstein-Barr virus (EBV), *Haemophilus influenzae* [16], [17], *Mycoplasma pneumoniae* [18] and influenza [19]–[23]. Recent work in England using time-series methods has identified associations between numbers of weekly reports of laboratory-confirmed infections with *Campylobacter*, *M. pneumoniae* and influenza, and incidence of hospitalization for GBS in subsequent weeks [24]. In the United States, influenza vaccination during 1976–1977 was associated with a seven-fold excess risk of GBS in the subsequent six-week period [25], and polio vaccination has been suggested as a risk factor for GBS in Finland and China [26], [27]. A recent study in England, however, found no association between any vaccination and subsequent GBS risk [28].

In order to better define the excess risk of GBS associated with these exposures, we undertook a nested case-control analysis in a United Kingdom-based general practice setting using data from the General Practice Research Database.

## Methods

### General Practice Research Database

The General Practice Research Database (GPRD) constitutes several hundred general practice clinics (GP clinics) serving a 5% representative sample of the UK population. The characteristics of this data source have been previously described [29]. The database holds electronic records of all patients registered with participating clinics, including basic patient information (birthdate, sex, registration and de-registration dates, death date) and records of all consultations with corresponding diagnoses, preventions (e.g. immunizations, screening) and prescriptions. Approval for the study was obtained from the scientific and ethics advisory group of the GPRD.

Data from participating clinics are validated to ensure accuracy and completeness for a minimum set of variables. Data meeting minimum quality criteria are termed ‘up-to-standard’ (UTS) data and are a general indicator of the overall quality of data from a given clinic [29]. Data from any clinic not meeting UTS criteria were excluded. We define an individual's up-to-standard follow-up time as the time during which they were registered with a clinic reporting UTS data.

GPRD diagnoses are recorded using Read or Oxmis (Oxford Medical Information Systems) codes, standardized terms used by medical practitioners to record patient outcome or management information, such as medical diagnoses, symptoms, test results and family history. We obtained information on all first consultations for GBS occurring between 1990 and 2001. A consultation is defined as any contact between a patient and the clinic services that appears in their medical records. We excluded repeat consultations. For these patients, we extracted all consultations for these infections: *Campylobacter*, cytomegalovirus, Epstein-Barr virus, *H. influenzae*, *M. pneumoniae* and influenza-like illness (ILI). We also included two sets of non-specific codes for infectious intestinal disease (IID) and acute respiratory infection (ARI) of unspecified aetiology. Finally, we obtained records of all influenza and polio vaccinations administered to GBS patients. A list of all diagnostic codes used is available from the authors.

Cases were defined as individuals with a first GBS consultation within their UTS follow-up time. Cases with under one year of UTS time available were excluded. We also excluded GBS consultations occurring within four months of patients' registration with their clinic or on the same day as a “new patient screening” to avoid inclusion of prevalent GBS recorded on joining a new clinic [30].

**Figure 1:**
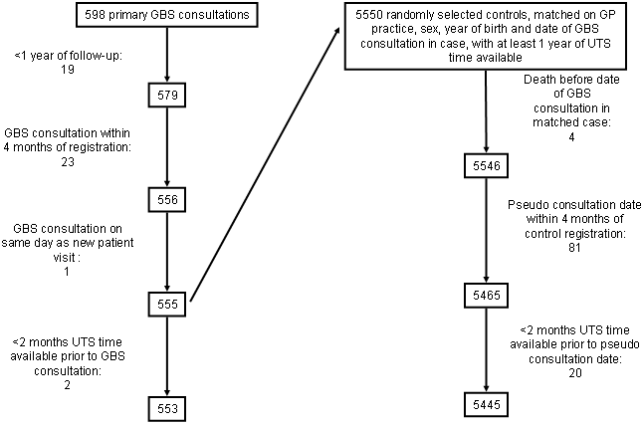
Case and control exclusions.

We randomly selected 10 controls per case, matched on GP clinic, sex, birth year (within one year for cases aged under 16 years or within five years otherwise) and GBS consultation date. We matched on the latter because the risk period for GBS following infection is short and some of the pathogens are highly seasonal. For each control, we assigned a pseudo-outcome date–the date of GBS consultation in their matched case. Controls were excluded if they had under one year of UTS time available or their pseudo-outcome date was within four months of clinic registration. Figure 1 presents details of case and control exclusions.

### Statistical analysis

**Figure 2:**
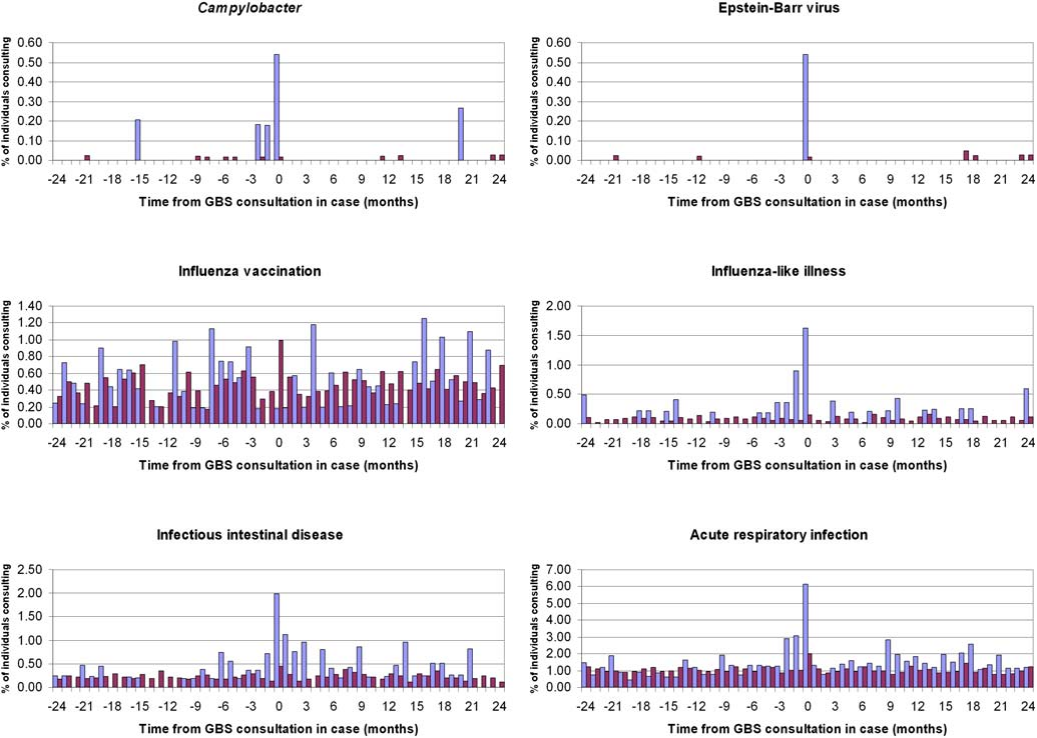
Incidence of consultation for various infections and influenza vaccination among GBS cases (open bars) and matched controls (dark bars) by time from GBS consultation in cases. X-axis - time from GBS consultation in cases or pseudo-outcome date in controls (months), y-axis - percentage of individuals consulting for infections or vaccination.

The risk period for GBS following infection is thought to be only a few weeks. Most GBS cases will be diagnosed in hospital emergency or neurology departments; the patient's general practitioner will be notified upon discharge, potentially several weeks or months after initial diagnosis. It is thus possible that there could be a considerable delay between a patient's initial consultation for neurological symptoms and the time when a confirmed GBS diagnosis is actually recorded. As this has implications for defining the exposure period, we first investigated the temporal relationship between infection and GBS in cases and controls. Figures 2a–f show incidences of consultation for the various infections in cases and controls by month from GBS consultation in cases. No consultations for cytomegalovirus, *H. influenzae* or *M. pneumoniae* were identified; these exposures were excluded from further analyses. From these figures, we defined the risk period as 60 days prior to GBS consultation in cases (or pseudo-outcome date in controls) and excluded individuals with incomplete UTS time during this period. If one such case was excluded, all their matched controls were excluded.

The final dataset comprised 553 cases and 5445 matched controls. We based power calculations on the ability to detect a significant difference in the prior two-month incidence of *Campylobacter* infection between cases and controls. We used data from a community-based cohort study of IID in England to estimate the two-month incidence of *Campylobacter* infection in the general population [31]. Assuming a conservative value for the between-sets correlation coefficient of 0.2, our study had 90% power to detect a minimum odds ratio of 10 at the 0.05 significance level.

We created indicator variables defining whether or not an individual had consulted for each of the exposures within the 60-day risk period. An individual was allowed to contribute only one consultation per condition during this period, resulting in a set of binary exposures.

We computed univariate matched odds ratios (ORs) and 95% confidence intervals (CIs) using conditional logistic regression. With the exception of one case with influenza-like illness who also received polio vaccine within the two-month exposure period, there were no individuals with multiple exposure events (two or more infections or vaccinations, or infection and vaccination); multivariable analysis was thus not performed. For GBS patients with preceding influenza-like illness or polio vaccination, for which there were sufficient numbers, we compared the median number of GP consultations for any condition in the 12 months following GBS diagnosis with that in the 12 months prior to GBS diagnosis using the Wilcoxon signed-rank test for matched pairs. All analyses were performed using Stata version 8.0 (StataCorp, Texas).

### Correction for under-ascertainment of Campylobacter infection

There is considerable under-ascertainment of infection in the GPRD: not all community *Campylobacter* enteritis cases present to general practice and, of those that do, only some are confirmed microbiologically. Recording of infections in patients' records may also be incomplete. The degree of under-ascertainment will vary between organisms, but is non-differential (independent of case or control status), as infection is diagnosed prior to GBS. Thus, the estimate of the OR will, on average, be biased towards the null; for a given infection incidence, the lower the ascertainment, the greater the bias [32]. For *Campylobacter* enteritis, the community incidence in England is estimated at 8.7 per 1000 personyears [31]. The incidence in the GPRD population is 0.5 per 1000 personyears. The ratio of GPRD to community incidences estimates the ascertainment probability for *Campylobacter* enteritis (0.058). We assume the specificity to be 1.0, as a culture-confirmed diagnosis of *Campylobacter* is unlikely to be false positive.

For a two-by-two table with *a* exposed cases, *b* exposed controls, *c* unexposed cases and *d* unexposed controls, the expected number of truly exposed cases is *a/s*, where *s* is the ascertainment probability. Similarly, *b/s* gives the expected number of truly exposed controls. By re-assigning the appropriate number of individuals from *c* to *a* and *d* to *b*, a corrected estimate of the true OR is obtained. This adjustment disregards matching and does not account for variability in ascertainment; bias could still arise if ascertainment probability is associated with the matching factors. The major factor influencing ascertainment is likely to be GP clinic, as diagnostic practices differ between clinics. We performed an OR correction by simulating 1000 matched case-control analyses in which the ascertainment probability for *Campylobacter* was fixed within clinics, regardless of case or control status, but allowed to vary between clinics. We assumed an underlying population of GP clinics with a true *Campylobacter* enteritis incidence of 8.7/1000 personyears. We then obtained GP clinic-specific incidences of *Campylobacter* enteritis using standard survival analysis methods similar to those in our previous GPRD study [14]. The ratio of these to the true incidence gives a distribution of ascertainment probabilities, *s*, across all clinics. The logarithm of this distribution is approximately Normal with mean −3.01 and standard deviation 0.76 (data not shown). For each clinic, we randomly assigned a value of *s* from this distribution. Within each clinic, we calculated the probability that an unexposed case actually had prior *Campylobacter* infection, (*a/s−a*)/*c*, based on this value of *s* and conditional values of *a* and *c* from the observed results (table 1, row 1). The corresponding probability for controls was calculated using the respective values for *b* and *d*. These two probabilities were applied to unexposed individuals within clinics to randomly re-assign exposure status among cases and controls, and an OR estimate obtained by conditional logistic regression. A thousand such simulations were performed to obtain the corrected OR distribution. The median OR and central 95% range of this distribution are presented. Comparable community incidence estimates were unavailable for other organisms; correction for under-ascertainment of these was not attempted.

**Table 1:** Distribution of preceding infections in GBS cases and controls, and univariate matched ORs and 95% CIs, General Practice Research Database, United Kingdom 1991–2001

	Cases (n = 553)		Controls = 5445			95% CI		
Exposures	No. exposed	%	No. exposed	%	Matched OR	Lower	Upper	p
*Campylobacter*	4	0.72	1	0.02	38.38	4.29	343.54	0.001
Epstein-Barr virus	2	0.36	1	0.02	20.00	1.81	220.56	0.014
Influenza-like illness	14	2.53	9	0.17	18.64	7.49	46.37	<0.001
Influenza vaccination	1	0.18	47	0.86	0.16	0.02	1.25	0.081
Polio vaccination	16	2.89	0	0.00	–	–*	–	
Infectious intestinal disease	13	2.35	18	0.33	7.26	3.52	14.99	<0.001
Acute respiratory infection	45	8.14	102	1.87	5.15	3.51	7.58	<0.001

* The exact conditional likelihood for was not estimable

### Population attributable fraction (PAF)

We estimated the proportion of GBS cases attributable to *Campylobacter* as:
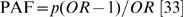
where *p* represents the *Campylobacter* enteritis prevalence among cases. We estimated *p* as the mean number of exposed cases across the 1000 simulations divided by the total number of cases.

## Results

Figures 2a–f show clearly elevated incidences of consultation for *Campylobacter*, EBV, ILI, IID and ARI in the two months prior to GBS consultation in cases compared with controls. No such difference is apparent for influenza vaccination.

Table 1 presents exposure distributions in cases and controls, matched ORs, 95% CIs and p-values. For polio vaccination, 16 instances of vaccination were identified among cases, but none among controls; a lower 95% confidence limit for the OR by exact likelihood methods, using the PROC LOGISTIC module in SAS version 9.1 (SAS Institute, North Carolina), was not estimable. The excess risk of GBS in the two months following *Campylobacter* infection, independent of GP clinic, sex, age and season, was 38-fold (OR = 38.4, 95% CI: 4.3–343.5); for EBV, the excess risk was 20-fold. Confidence intervals are wide, reflecting the small number of these infections identified. Influenza-like illness carried an 18-fold increase in GBS risk (OR = 18.6, 95% CI: 7.5–46.4); for IID and ARI, the excess risks were seven- and five-fold respectively. Influenza vaccination appeared protective (OR = 0.16, 95% CI: 0.02–1.25), although this result was not significant at the 0.05 level of precision.

Among GBS patients with influenza-like illness, there were a total of 256 GP consultations for any condition in the 12 months following GBS diagnosis, compared with 145 in the 12 months prior to GBS. The difference in the median number of presentations between these two time periods was highly significant (post-GBS median = 16, interquartile range (IQR): 10–26; pre-GBS median = 10, IQR: 4–11, Wilcoxon signed-rank z = 3.13, p = 0.0017). No such difference was found for GBS cases with preceding polio vaccination.

### Corrected Campylobacter estimates

**Figure 3:**
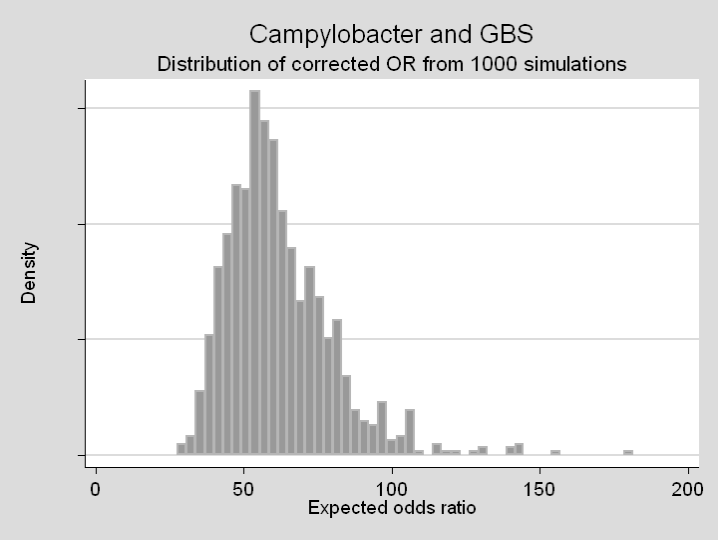
Distribution of corrected OR for the association between *Campylobacter* enteritis and GBS, based on 1000 simulations with varying values of clinic-level ascertainment of *Campylobacter* infection (see text).

With a fixed under-ascertainment probability of 0.058, the corrected OR was 44.8. The corrected estimate allowing for clinic-level matching and ascertainment variability yielded an OR distribution with median 58.7 and central 95% range 36.9–105.2 (figure 3). One fifth of GBS cases were attributable to *Campylobacter* (PAF = 20.1%).

## Discussion

We have found strong, positive associations between infection with *Campylobacter*, EBV and ILI and GBS risk. Specifically, individuals with *Campylobacter* enteritis are 38 times more likely to be diagnosed with GBS in the subsequent two months, and our correction for under-ascertainment of *Campylobacter* infection suggests that the excess risk could be 60-fold. Approximately 20% of GBS cases are attributable to *Campylobacter* infection.

Our results also provide evidence for an 18-fold increased risk of GBS in the two months following ILI. Such a strong, quantitative association with ILI has not previously been reported. Given the high incidence of influenza during epidemic seasons, the burden of GBS attributable to this organism could be substantial. We found some evidence of significant excess use of primary care services in the 12 months following GBS diagnosis among patients with preceding ILI, with a median of six excess consultations compared with the 12 month period preceding GBS diangosis. However, this does not take into account the nature of the consultations and may not reliably reflect the extent of excess healthcare use, since many GBS cases may receive follow-up care at hospital outpatient departments, and these consultations will not appear on their primary care records. It should be noted that our estimate of the excess risk of GBS following ILI will be influenced both by under-ascertainment of true influenza and inclusion of false-positive influenza, as most cases are not virologically confirmed. In both these situations, the OR will, on average, be under-estimated; the true OR could be higher than that observed. As influenza incidence varies greatly both within and between years, the PAF for this organism will vary over time. The lower ORs for IID and ARI are not surprising, as most of these infections will be caused by pathogens unrelated with GBS.

Unlike previous reports, we found evidence suggesting a protective effect of influenza vaccination on GBS risk. This finding is biologically plausible–influenza vaccination provides some protection against influenza infection and, hence, associated complications. It is, however, also possible that the protective effect of influenza vaccination is a result of individuals suffering a recent acute infection being less likely to be offered vaccine. As there were no individuals who both suffereed ILI and received vaccine within the exposure period, we were unable to investigate this possibility. Historical cohort studies of primary care data comparing GBS risk among vaccinees and non-vaccinees would be better suited to address this. The apparent protective effect of vaccine found here is not inconsistent with an absolute increase in GBS risk following influenza vaccination, but indicates that this is much smaller than the risk associated with influenza infection. The balance between GBS risk and protection from vaccine will reflect the frequency of influenza in a given season. The exact vaccine formulation could also influence risk. An association with polio vaccination was also apparent, although it should be noted that while most such vaccinations take place among young children, the majority of GBS cases occur in older individuals. It is thus unlikely that polio vaccination accounts for a substantial proportion of GBS cases in the UK. Historical cohort studies, as described above for influenza vaccination, could yield further insight into the association between GBS and polio vaccination, and could in addition investigate the effect of vaccine dosage.

Our study has certain limitations. Individuals consulting general practice are symptomatic. Evidence suggests that, at least for *Campylobacter*, GBS may arise following asymptomatic infection [34]; we could not address this in our study. Other than age and sex, GPRD data contain little information on potential confounders, such as geographic location and socioeconomic status; we addressed this by matching on GP clinic. Due to under-ascertainment of infections, our estimates are likely to be biased towards the null. For *Campylobacter* enteritis, for which a reliable estimate of community incidence exists, we corrected for this by accounting for the magnitude and variability of ascertainment at clinic level, the factor most likely to influence ascertainment. Some residual bias might remain through ascertainment differences by sex and/or age. Although such residual bias could affect the magnitude of bias in the OR, it is unlikely to affect its direction unless ascertainment is differential (dependent on case status) in one or more age/sex strata. We think this highly unlikely, as in our study infection was determined before GBS. Our correction additionally assumes that clinic-level variation in *Campylobacter* incidence is entirely due to ascertainment differences rather than differences in true incidence. These two factors cannot be disentangled without knowledge of the variation in true *Campylobacter* incidence across clinics. Given the low ascertainment in all GP clinics, we believe this assumption is reasonable.

Our analysis has several advantages over previous GBS risk factor studies. Firstly, we used a representative sample of cases and controls from the UK population; our results are more generalizable than those of studies conducted in hospital settings. Secondly, although our study suffered from under-ascertainment of infections, this was non-differential. By contrast, exposure misclassification in studies using serology to determine prior *Campylobacter* infection cannot be expected to be non-differential. Evidence suggests that following *Campylobacter* infection, antibody levels remain elevated for several months and even years [35]–[37]. The temporal association between *Campylobacter* and GBS means that seropositivity in GBS cases is more likely to indicate true recent infection, while in controls it could signify recent infection, past infection, or immunity. Such studies thus overestimate the incidence of recent infection in controls relative to cases, yielding ORs substantially below those observed in our study.

Our findings indicate the value of primary care data for studying rare complications of infectious diseases. Although ascertainment of infections can be low, figure 2 clearly demonstrates that such systems are sensitive for detecting temporal associations between infection and sequelae. We recommend more widespread use of such systems for surveillance purposes. The data are routinely available, considerably cheaper than the operating costs for a dedicated surveillance system, and particularly advantageous for rare conditions. There are also considerable advantages for observational studies; cases are effectively nested within a cohort, minimizing the risk of selection, recall and diagnostic biases, common problems in conventional case-control studies.

Using this strategy, we have detected two novel findings: an increased risk of GBS following ILI and a possible protective effect of influenza vaccination. Clinicians should consider recent history of influenza as a possible triggering factor in GBS cases. Our findings also suggest that influenza vaccination may provide additional, indirect effects through protection against complications of influenza infection. Further studies to determine the incidence of GBS following both vaccination against and infection with influenza are warranted.
